# TRAF6 Autoubiquitination-Independent Activation of the NFκB and MAPK Pathways in Response to IL-1 and RANKL

**DOI:** 10.1371/journal.pone.0004064

**Published:** 2008-12-29

**Authors:** Matthew C. Walsh, Gregory K. Kim, Paul L. Maurizio, Elizabeth E. Molnar, Yongwon Choi

**Affiliations:** Department of Pathology and Laboratory Medicine, University of Pennsylvania School of Medicine, Philadelphia, Pennsylvania, United States of America; New York University School of Medicine, United States of America

## Abstract

The adapter protein TRAF6 is critical for mediating signal transduction from members of the IL-1R/TLR and TNFR superfamilies. The TRAF6 RING finger domain functions as an ubiquitin E3 ligase capable of generating non-degradative K63-linked ubiquitin chains. It is believed that these chains serve as docking sites for formation of signaling complexes, and that K63-linked autoubiquitination of TRAF6 is essential for formation and activation of a complex involving the kinase TAK1 and its adapters, TAB1 and TAB2. In order to assess independently the E3 ligase and ubiquitin substrate functions of TRAF6, we generated, respectively, RING domain and complete lysine-deficient TRAF6 mutants. We found that while the TRAF6 RING domain is required for activation of TAK1, it is dispensable for interaction between TRAF6 and the TAK1-TAB1-TAB2 complex. Likewise, lysine-deficient TRAF6 was found to interact with the TAK1-TAB1-TAB2 complex, but surprisingly was also found to be fully competent to activate TAK1, as well as NFκB and AP-1 reporters. Furthermore, lysine-deficient TRAF6 rescued IL-1-mediated NFκB and MAPK activation, as well as IL-6 elaboration in retrovirally-rescued TRAF6-deficient fibroblasts. Lysine-deficient TRAF6 also rescued RANKL-mediated NFκB and MAPK activation, and osteoclastogenesis in retrovirally-rescued TRAF6-deficient bone marrow macrophages. While incapable of being ubiquitinated itself, we demonstrate that lysine-deficient TRAF6 remains competent to induce ubiquitination of IKKγ/NEMO. Further, this NEMO modification contributes to TRAF6-mediated activation of NFκB. Collectively, our results suggest that while TRAF6 autoubiquitination may serve as a marker of activation, it is unlikely to underpin RING finger-dependent TRAF6 function.

## Introduction

Non-conventional K63-linked ubiquitination has gained attention in recent years as a mechanism of protein scaffold building for complex formation during signal transduction. Upon activation, HECT or RING finger domain-containing E3 ubiquitin ligases affix these non-degradative ubiquitin chains to protein substrates, which then serve as recruitment platforms for downstream signaling mediators [1], [2]. The close proximity of factors generated by these scaffolds is believed to promote otherwise energetically inefficient conformational adaptations or phosphorylation events [1], [2], [3]. A particularly notable K63-specific RING finger E3 ubiquitin ligase is TNFR-associated factor 6 (TRAF6), which was identified as having such activity upon being isolated from an IκB kinase (IKK)-activating complex [4], [5]. Like most other TRAFs, TRAF6 is composed of an N-terminal RING finger domain, a series of zinc fingers, a coiled-coil domain, and a C-terminal TRAF domain. TRAF6 is unique from other TRAFs in that it utilizes a distinct interaction motif, which is found in its upstream activators [6]. As such, TRAF6 has been implicated in directing the signals from representative members of a diverse array of receptor families, including the TNF receptor superfamily (TNFRSF), IL-1R/Toll-like receptor superfamily (IL-1R/TLRSF), TGFβR, IL-17R and IL-25R, and the NOD-like pattern recognition receptors [7], [8], [9], [10], [11], [12], [13]. TRAF6 activation leads to downstream activation of PI3K, the mitogen-activated protein kinase (MAPK) cascade, and the transcription factor families NFκB, NFAT, and IRF [12], [14], [15]. Numerous biochemical and genetic studies have revealed mechanistic insights into TRAF6 utilization of ubiquitination to propagate diverse signals. To date, the most prominent model holds that upon activation via TRAF6 homo-oligimerization, the RING finger ubiquitin E3 ligase domain complexes with a K63-specific E2 conjugating enzyme (Ubc13/Uev1a, or possibly UbcH7) to mediate attachment of non-degradative K63-linked ubiquitin chains to TRAF6 substrates, specifically TRAF6 itself [4], [16]. These chains recruit factors, like the adapters TAB2/3, which contain atypical zinc finger domains with a special affinity for binding K63-linked ubiquitin chains [17]. As part of a pre-formed complex, TAB2/3 bring TAB1 and the putative IKK and MKK kinase TAK1 to TRAF6 [5]. It is believed that TAK1 is then activated via a trans-phosphorylation event made possible by the close proximity generated by complex formation [1], [18]. Activated TAK1 then phosphorylates downstream factors. However, the complexities involved in studying ubiquitin-mediated signaling events have made certain aspects of this model difficult to formally demonstrate. For instance, putative ubiquitin targets are often ubiquitinated with both K48-linked degradative and K63-linked non-degradative chains at multiple locations. A further complication is the propensity of ubiquitination complexes to generate chains of varying lengths at a given modification site *in vivo*, making detection of ubiquitination by traditional size separation techniques challenging. Attempts to genetically manipulate lysine acceptor sites on protein substrates with the intention of ablating E3 ligase-specific ubiquitination may result in dramatically decreased modification, while failing to entirely eliminate physiologically relevant ubiquitin chains that happen to be affixed at low levels or heterotypic lengths. Lastly, while autoubiquitination is often readily detected in the context of *in vitro* ubiquitination, these assays feature a well-recognized caveat of increased E3 ligase promiscuity in the absence of alternative targets, such that the same autoubiquitination event observed *in vitro* may not have physiologic relevance *in vivo* [19], [20]. Therefore, we sought to investigate, through genetic approaches, the relationship between TRAF6 RING finger ubiquitin E3 ligase activity, TRAF6 (auto)ubiquitination (for the purpose of clarity we will refer to all K63-linked ubiquitination of TRAF6 as autoubiquitination, while recognizing that additional ubiquitin E3 ligases may be involved in TRAF6 K63-linked modification *in vivo*) and recruitment and activation of downstream signaling. We found that while the TRAF6 RING finger domain is required for activation of TAK1, it is dispensable for interaction between TRAF6 and the TAK1-TAB1-TAB2 complex. Surprisingly, we found that TRAF6 autoubiquitination was dispensable for both interaction with, and activation of, the TAK1 signaling complex. Further, TRAF6 autoubiquitination, but not RING finger function, was found to be dispensable for IL-1- or RANKL-dependent elaboration of IL-6, or osteoclastogenesis, respectively. Finally, TRAF6 autoubiquitination was found not to be required for TRAF6 to ubiquitinate an alternative target, specifically IKKγ (NEMO).

## Results

### TRAF6 associates with the TAK1-TAB1-TAB2 complex in the absence of a functional TRAF6 RING finger domain

**Figure 1:**
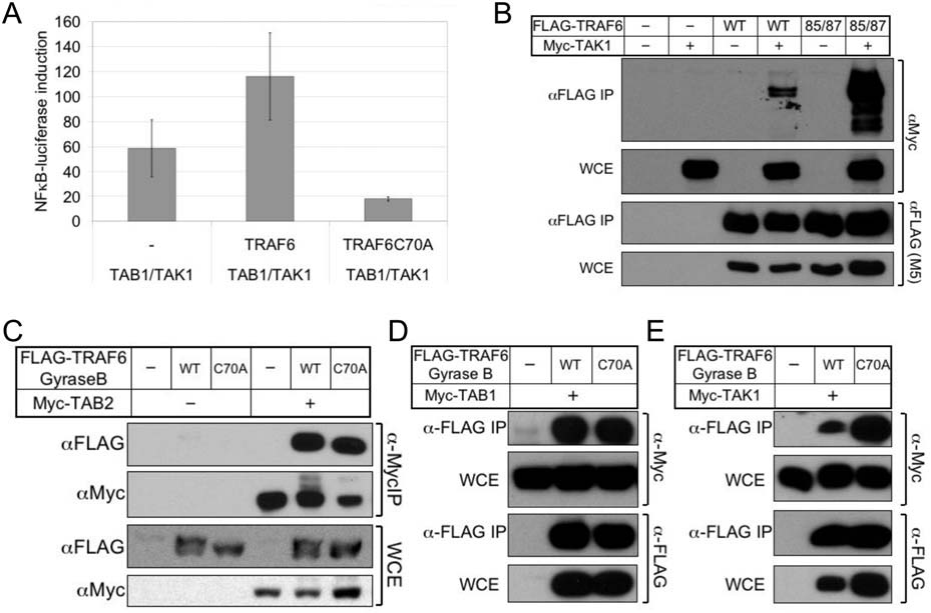
TRAF6 associates with the TAK1-TAB1-TAB2 complex in the absence of a functional TRAF6 RING finger domain. (A) 293T cells were transfected with NFκB-luciferase, 40 ng TAB1 and TAK1, and 400 ng of empty vector (EV), TRAF6, or TRAF6 C70A as indicated. Cell lysates were analyzed by luciferase reporter assay. Values indicate fold increase over background and are normalized against a β-galactosidase internal standard. (B) 293T cells were transfected with equal amounts (1 µg) of epitope-tagged TRAF6 (FLAG) or TAK1 (Myc) expression plasmids and subjected to immunoprecipitation (IP) with anti-FLAG agarose. IP and whole cell extracts (WCE) were immunoblotted with anti-Myc or anti-FLAG as indicated. (C), (D), (E) 293T cells were transfected with WT or C70A FLAG-TRAF6(1-358)-Gyrase B as indicated, plus an equal amount of Myc-tagged TAB2 (C), TAB1 (D) or TAK1 (E). Cell lysates were processed and analyzed as in (B).

It has previously been demonstrated that TRAF6 RING finger function, K63-linked ubiquitination, and the ubiquitin-binding domain of TAB2 are required for TRAF6-mediated activation of downstream signaling pathways [17]. It is believed that TRAF6 autoubiquitination serves as a point of recruitment for TAB2, and hence TAB1 and TAK1. If so, we reasoned that overexpression of a TRAF6 mutant possessing a defective RING finger (C70A), and therefore incapable of autoubiquitination, should not inhibit NFκB activation induced by co-overexpression of TAK1 and its activator, TAB1. Rather, in reporter assays for NFκB, we found that while overexpression of wild-type TRAF6 in conjunction with TAB1 and TAK1 enhanced reporter activity over TAB1 and TAK1 alone, overexpression of RING finger-mutant TRAF6 (C70A) caused a greater than 2-fold reduction in activity (Fig. 1A). To assess the importance of the TRAF6 RING finger function to interaction with the TAK1 complex, we performed a co-immunoprecipiation assay, and found that another TRAF6 mutant containing a defective RING finger (C85A/H87A) interacted more strongly with TAK1 than did wild-type TRAF6 (Fig. 1B). Like other TRAF proteins, TRAF6 forms homo- and heterotypic interactions via a C-terminal TRAF domain [21]. To exclude the possibility that a full-length TRAF6 RING mutant might compensate for defective complex formation through interactions with endogenous TRAF proteins, we generated mutants in which the C-terminus of TRAF6 is replaced with the artificial oligomerization domain of bacterial Gyrase B. Again, we found that RING-mutant TRAF6 interacted as well, or better with TAB2 (Fig. 1C), TAB1 (Fig. 1D), and TAK1 (Fig. 1E). Given that TRAF6 autoubiquitination requires a functional RING finger, these observations challenge the putative relationship between TRAF6 autoubiquitination and recruitment of the TAK1 signaling complex to TRAF6.

### A lysine-deficient TRAF6 N-terminus-Gyrase B fusion protein interacts with and activates TAK1, and induces NFκB and AP-1 reporter activity

**Figure 2:**
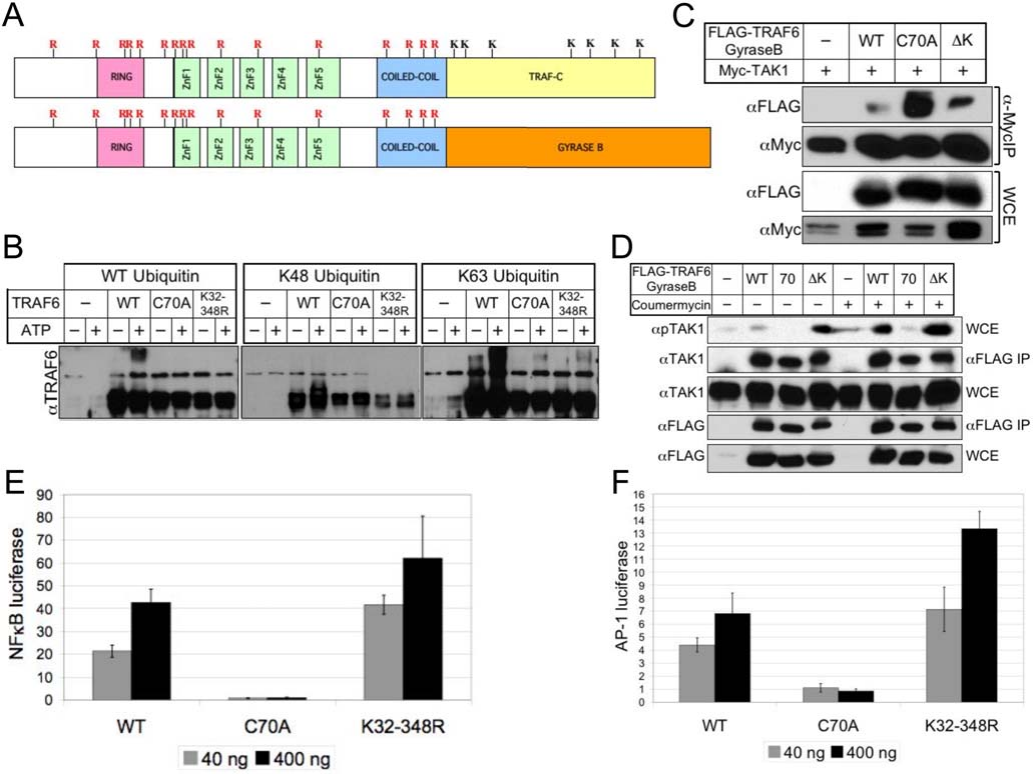
A lysine-deficient TRAF6 N-terminus-Gyrase B fusion protein interacts with and activates TAK1, and induces NFκB and AP-1 reporter activity. (A) Generation of mutant versions of murine TRAF6 containing lysine (K) to arginine (R) mutations in the RING finger, zinc finger, and coiled-coil domains (TRAF6 K32-348R) (top), or in the RING finger, zinc finger, and coiled-coil domains, but with the TRAF6-C domain replaced with the Gyrase B artificial oligomerization domain (TRAF6(1-358)ΔK-Gyrase B) (bottom). (B) 293T cells were transfected with the indicated full-length versions of FLAG-TRAF6, then lysates were immunoprecipitated with FLAG and subjected to *in vitro* ubiquitination in the presence or absence of ATP using recombinant WT, K48-only, or K63-only ubiquitin. Unmodified and modified TRAF6 were detected by immunoblotting with anti-TRAF6. (C) 293T cells were transfected with equal amounts (1 µg) of epitope-tagged TAK1 (Myc) in combination with either wild-type (WT), RING mutant (C70A), or lysine-deficient (ΔK) TRAF6(1-358)-Gyrase B (FLAG) expression plasmids, and subjected to immunoprecipitation (IP) with anti-FLAG agarose. IP and whole cell extracts (WCE) were immunoblotted with anti-Myc or anti-FLAG as indicated. (D) 293T cells were transfected with 10 ng of TAK1 in combination with either wild-type (WT), RING mutant (C70A), or lysine-deficient (ΔK) TRAF6(1-358)-Gyrase B (FLAG) expression plasmids, and left untreated or treated for 5 minutes with Coumermycin A before being harvested and subjected to IP with anti-FLAG agarose. IP and whole cell extracts (WCE) were immunoblotted with anti-phospho TAK1 (T187), anti-TAK1, or anti-FLAG as indicated. (E), (F) 293T cells were transfected with NFκB-luciferase (E) or AP-1-luciferase (F) plus WT, C70A, or K32-348R TRAF6(1-358)-Gyrase B as indicated. Cell lysates were analyzed by luciferase reporter assay. Values indicate fold increase over background and are normalized against a β-galactosidase internal standard.

Like many E3 ubiquitin ligases, TRAF6 has the capacity to autoubiquitinate *in vitro* [4], (Fig. S1). Physiologic relevance of TRAF6 autoubiquitination has been inferred from the dual observations that 1) TRAF6 autoubiquitination is detected *in vivo* upon engagement of TRAF6-dependent receptors, and 2) the TRAF6 RING finger is required for propagating downstream signal transduction. Recent efforts have been made to map the lysines residue(s) on TRAF6 targeted during *in vitro* autoubiquitination [22]. However, it remains unclear whether, or in what pattern, TRAF6 might combine with other K63-specific E3 ubiquitin ligases to ubiquitinate TRAF6 *in vivo*. Therefore, we sought to divorce the RING finger function of TRAF6 from its capacity to serve as a substrate for ubiquitination. We employed a mutagenesis approach targeting individual lysine residues, which form isopeptide bonds with the carboxyl-terminal glycine 76 of ubiquitin, to ablate TRAF6 autoubiquitination during *in vivo* overexpression. However, we were only able to achieve partial reduction in ubiquitination, possibly due to modification of TRAF6 at multiple sites. Therefore, reasoning that we could focus on the N-terminus, due to its ability to activate downstream signaling when fused to Gyrase B, we simultaneously mutated all N-terminal TRAF6 lysine residues (K32-348) on both full-length and (1-358)-Gyrase B fusion versions of TRAF6 (Fig. 2A). We tested the ability of the full-length TRAF6 (K32-348R) mutant to autoubiquitinate in an *in vitro* ubiquitination assay and found that K63-specific modification of TRAF6 was reduced to a level similar to that of the TRAF6 RING finger mutant (C70A) (Fig. 2B). By utilizing the TRAF6(1-358)-Gyrase B mutant (ΔK) to assess interaction with TAK1 in 293T cells, we found that ΔK interacted with TAK1 more strongly than wild-type TRAF6, though less strongly than C70A (Fig. 2C), demonstrating dispensability of TRAF6 autoubiquitination for complex formation with TAK1. In the IL-1 signaling pathway, optimal TAK1-mediated activation of JNK and NFκB requires phosphorylation of TAK1 at T187 (Fig. S2). To assess the ability of TRAF6 mutants to activate TAK1, we transfected low levels of TRAF6(1-358)-Gyrase B mutants and induced oligomerization using the drug Coumermycin A1. While all TRAF6 constructs exhibited interaction with TAK1 in the absence of drug, and only the RING mutant failed to activate TAK1 in the presence of drug, the ΔK mutant exhibited enhanced activation of TAK1 in comparison to wild-type TRAF6, even in the absence of drug treatment (Fig. 2D), suggesting that TRAF6 autoubiquitination may be dispensable for activation of TAK1-mediated signaling. The various TRAF6-Gyrase B mutants were then tested in reporter assays for NFκB and AP-1, and consistent with its apparent enhanced ability to activate TAK1, the ΔK mutant induced both NFκB (Fig. 2E) and AP-1 (Fig. 2F) reporters to higher levels than wild-type TRAF6.

### A lysine-deficient mutant TRAF6 rescues IL-1-mediated NFκB and MAPK activation, as well as IL-6 production, in TRAF6-deficient fibroblasts

**Figure 3:**
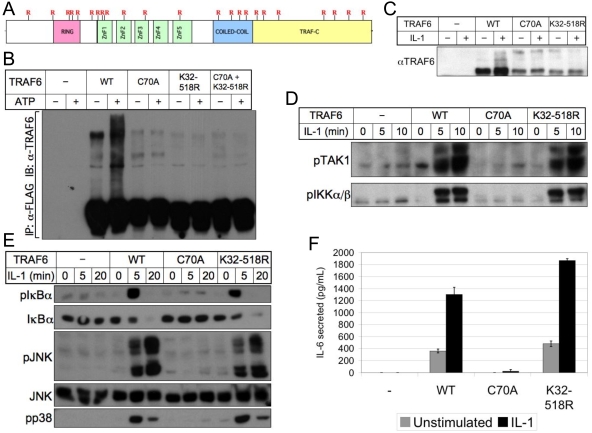
A lysine-deficient mutant TRAF6 rescues IL-1-mediated NFκB and MAPK activation, as well as IL-6 production, in TRAF6-deficient fibroblasts. (A) A mutant version of full-length TRAF6 containing lysine (K) to arginine (R) mutations at all lysine residues (referred to as TRAF6 K32-518R or TRAF6 ΔK) was generated, and mutations are depicted in relation to TRAF6 domain location. (B) TRAF6-deficient fibroblasts were retrovirally-rescued with the indicated full-length versions of FLAG-TRAF6, then lysates immunoprecipitated with FLAG and subjected to *in vitro* ubiquitination in the presence or absence of ATP using recombinant K63-only ubiquitin. The last two lanes represent C70A and K32-518R lysates mixed 1∶1 prior to immunoprecipitation to assess trans-ubiquitination potential of TRAF6 monomers. Unmodified and modified TRAF6 were detected by immunoblotting with anti-TRAF6. (C) Cell lines used in (B) were treated for 5 minutes with IL-1β, then lysed in the presence of N-ethyl maleimide (NEM) and subjected to immunoblotting with anti-TRAF6 to detect both unmodified and high molecular weight forms of TRAF6. (D), (E) TRAF6-deficient fibroblasts retrovirally-rescued with the indicated full-length versions of FLAG-TRAF6 were treated as indicated with IL-1β, then lysed and subjected to immunoblotting against the activated phosphorylated forms of TAK1 (D), IKKα/β (D) IκBα (E), JNK (E), and p38 (E). (F) TRAF6-deficient fibroblasts retrovirally-rescued with the indicated full-length versions of FLAG-TRAF6 were left untreated or treated for 12 hours with IL-1β. Supernatants were collected and assayed by ELISA for IL-6 production. Values are normalized to crystal violet assays of the cell culture plates.

To assess the requirement for TRAF6 autoubiquitination during physiologic receptor signaling, we generated TRAF6-deficient fibroblast cell lines retrovirally-rescued with either FLAG-tagged wild-type, RING finger mutant (C70A), or total lysine-deficient (K32-518R) TRAF6 (Fig. 3A). Anti-FLAG immunoprecipitation of these proteins was carried out, followed by an *in vitro* ubiquitination assay which demonstrated not only that neither C70A nor K32-518R could autoubiquitinate, but also that a mixture of the two was incapable of autoubiquitination in trans (Fig. 3B), suggesting the importance of TRAF6 oligimerization for E3 ligase activity. IL-1R signaling in TRAF6-deficient fibroblasts is completely defective. *In vivo*, induction of high molecular weight TRAF6, in response to IL-1, treatment was detected in fibroblast cell lines expressing wild-type, but not C70A or K32-518R versions of TRAF6 (Fig. 3C), though *in vivo* expression levels of TRAF6 K32-518R were consistently much lower than those of wild-type TRAF6. Regardless, TRAF6 K32-518R was capable of activating the kinases TAK1 and IKK in response to IL-1 in a manner similar to wild-type TRAF6 (Fig. 3D). IL-1-dependent activation of the MAPKs JNK and p38, and phosphorylation and degradation of the NFκB inhibitor IκBα were also normal in TRAF6 K32-518R-rescued cells (Fig. 3E). Though activation of these key downstream signaling pathways in the absence of TRAF6 autoubiquitination appeared normal, TRAF6-mediated gene regulation involves complex coordination of multiple transcription factor families, leaving the possibility that genetic regulation by TRAF6 K32-518R might be defective. To address this possibility, we assayed elaboration of the inflammatory cytokine IL-6 by retrovirally-rescued fibroblasts in response to IL-1 treatment, and found that while TRAF6 C70A failed to induce IL-6 production, TRAF6 K32-518R-rescued cells produced IL-6 at slightly higher levels than cells rescued with wild-type TRAF6 (Fig. 3F).

### Lysine-deficient mutant TRAF6 rescues RANKL-mediated NFκB and MAPK activation, as well as osteoclastogenesis in TRAF6-deficient BMM

**Figure 4:**
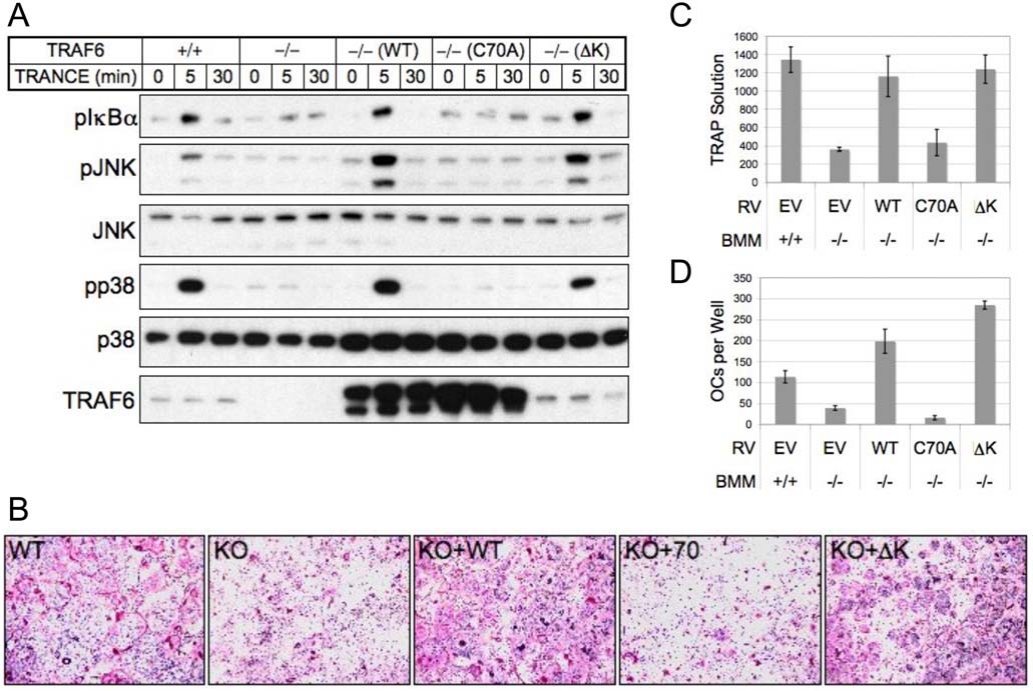
A lysine-deficient mutant TRAF6 rescues RANKL-mediated NFκB and MAPK activation, as well as osteoclastogenesis in TRAF6-deficient BMM. (A) Wild-type or TRAF6-deficient bone marrow macrophages (BMM) retrovirally-rescued with wild-type (WT), RING mutant (C70A), or lysine-deficient (ΔK) full-length versions of FLAG-TRAF6 were treated as indicated with RANKL, then lysed and subjected to immunoblotting against the activated phosphorylated forms of IκBα, JNK, and p38. *B*, BMM described in (A) were replated and cultured with M-CSF and RANKL for 5 days to induce osteoclast differentiation. (C) Osteoclasts depicted in (B) were fixed and subjected to TRAP solution assay and quantified at 405 nm absorbance. (D) Total cell counts per well of retrovirally-rescued osteoclasts depicted in (B) as defined by cells containing at least 3 nuclei and being at least 100 µM in diameter.

In addition to mediating IL-1R/TLRSF signaling via interactions with IRAK, TRAF6 propagates TNFRSF signaling through direct receptor binding. To examine the role of TRAF6 autoubiquitination in TNFRSF signaling, we generated retrovirally-rescued TRAF6-deficient bone marrow macrophages expressing TRAF6 K32-518R, and treated with the TNFSF member RANKL. Again, we found that lysine-deficient TRAF6 was capable of activating the NFκB, JNK, and p38 pathways (Fig. 4A), despite considerably diminished protein expression. RANKL treatment *in vitro* is sufficient for BMM to develop into osteoclasts in a TRAF6-dependent manner [23]. We found that TRAF6-deficient BMM rescued with K32-518R, but not C70A were capable of undergoing osteoclastogenesis similarly to wild-type-rescued BMM (Fig. 4B). This observation was confirmed quantitatively by assaying expression of the osteoclast marker tartrate resistant acid phosphatase (TRAP) (Fig. 4C) and total counts of large multi-nucleated cells (Fig. 4D). These data confirm that TRAF6 can mediate a complex biological process in the absence of TRAF6 autoubiquitination.

### Lysine-deficient TRAF6 N-terminus-Gyrase B fusion protein is competent to mediate TRAF6-specific ubiquitin modification of NEMO

**Figure 5:**
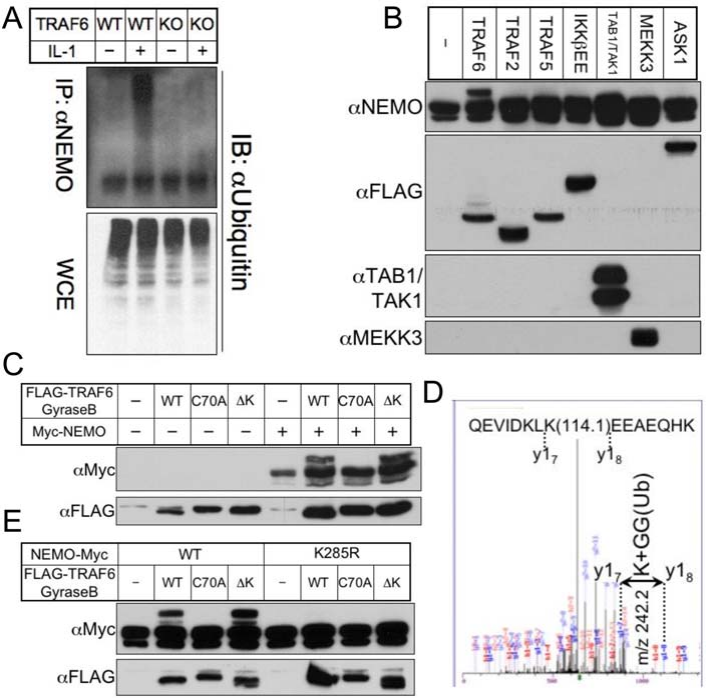
A lysine-deficient TRAF6 N-terminus-Gyrase B fusion protein remains competent to mediate a TRAF6-specific ubiquitin modification of NEMO. (A) Wild-type (WT) and TRAF6-deficient (KO) fibroblasts were stimulated for 5 minutes with IL-1β, then lysed with buffer containing NEM. To determine specificity of *in vivo* ubiquitination, lysates were dissociated and denatured before being immunoprecipitated with anti-NEMO. WCE and IP extracts were subjected to immunoblotting with anti-Ubiquitin. (B) 293T cells were transfected with equal amounts (1 µg) of Myc-tagged NEMO and expression plasmids for TRAF6, TRAF2, TRAF5, constitutively active IKKβ (IKKβEE), TAB1 and TAK1, MEKK3, or ASK1. Cells were lysed in the presence of phosphatase inhibitors and NEM, and WCE were then immunoblotted with anti-NEMO to detect modification of NEMO. (C) 293T cells were transfected with equal amounts (1 µg) of Myc-tagged NEMO and expression plasmids for either wild-type, RING mutant (C70A), or lysine-deficient FLAG-TRAF6(1-358)-Gyrase B. Cells were lysed in the presence of phosphatase inhibitors and NEM, and WCE were then immunoblotted with anti-NEMO to detect modification of NEMO. (D) 293T cells were transfected with 10 µg Myc-NEMO and co-transfected with wild-type or C70A TRAF6(1-358)-Gyrase B (10 µg). These plates, and an untransfected plate, were lysed and immunoprecipitated with anti-Myc agarose. IP extracts were dissociated from anti-Myc agarose by heating (65°C) in non-reducing SDS-PAGE buffer, and separated from agarose by centrifugation. Reducing agent was added to IP extracts, and 10% of each was used for immunoblotting with anti-Myc to confirm the presence of NEMO modification (not shown), while 90% was run on a preparative gel and stained with colloidal coomassie blue (not shown). The NEMO modification band found in the lane co-transfected with wild-type, but not with C70A TRAF6(1-358)-Gyrase B, was excised, digested with trypsin, and analyzed by mass spectrometry. Depicted is the m/z profile of the NEMO peptide QEVIDKLKEEAEQHK, for which all detected ions gave predicted values except for the fragment encompassing lysine 285 between y_7_ and y_8_, which exhibits a residue mass of 242.2. This profile is consistent with one lysine and two glycines, and is indicative of an ubiquitin modification at NEMO K285. (E) 293T cells were transfected with equal amounts (1 µg) of Myc-tagged wild-type or K285R NEMO in combination with expression plasmids for either wild-type, RING mutant (C70A), or lysine-deficient FLAG-TRAF6(1-358)-Gyrase B. Cells were lysed in the presence of phosphatase inhibitors and NEM, and WCE were then immunoblotted with anti-NEMO to detect modification of NEMO.

Substantial evidence exists for the general role of K63-linked ubiquitination in promoting TRAF6-mediated signal transduction, and while the TRAF6 RING finger domain is essential, the dispensability of TRAF6 autoubiquitination opened the possibility that the critical function of the RING domain is unrelated to ubiquitin E3 ligase activity. Therefore, we sought to identify an example of TRAF6 RING-mediated ubiquitination that occurs independently of TRAF6 autoubiquitination. IKKγ, the IKK regulatory component also known as NEMO, is ubiquitinated in response to TRAF6-dependent IL-1R/TLRSF stimulation [7], and has been shown to be a K63-linked target of TRAF6 E3 ligase activity in *in vitro* ubiquitination assays [22]. Consistent with these observations, we found that upon treatment with IL-1, NEMO immunoprecipitated from wild-type, but not TRAF6-deficient fibroblasts, was ubiquitinated (Fig. 5A). To determine the specificity of the NEMO modification, we co-transfected NEMO with either TRAF6, or various TRAF6-associated factors (TRAF2, TRAF5, constitutively-active IKKβ (IKKβEE), TAK1/TAB1, MEKK3, ASK1) known to act downstream of, or parallel to, TRAF6-dependent signaling pathways [24], [25], [26], [27], [28]. We found that despite the association of these various factors with NFκB reporter activation, only TRAF6 co-expression induced this distinct modification of NEMO (Fig. 5B). Having identified a clear TRAF6-inducible modification of NEMO, we sought to determine whether it required either the TRAF6 RING finger domain or ubiquitination of TRAF6. Using co-expression of NEMO with TRAF6(1-358)-Gyrase B fusion constructs containing either RING (C70A) or total lysine (ΔK) mutations, we found that while the RING finger was required for NEMO modification, TRAF6 autoubiquitination was dispensable. To confirm that the modification identified was in fact ubiquitination, we excised the modified band from a preparative gel and subjected it to nanoLC-MS/MS mass spectrometry, and identified an m/z signature consistent with ubiquitination at NEMO K285 (Fig. 5D), which is consistent with previous reports [7], [29]. We generated a NEMO K285R mutant and compared TRAF6-mediated modification of NEMO in the presence or absence of lysine 285. Confirming the likely role of TRAF6 in ubiquitinating NEMO independently of TRAF6 autoubiquitination, we found that co-expression of either wild-type TRAF6 or TRAF6 ΔK with NEMO K285R resulted in a complete abrogation of the TRAF6-dependent modification observed on wild-type NEMO (Fig. 5E).

### TRAF6-associated ubiquitination of NEMO is required for optimal IL-1-mediated activation of NFκB

**Figure 6:**
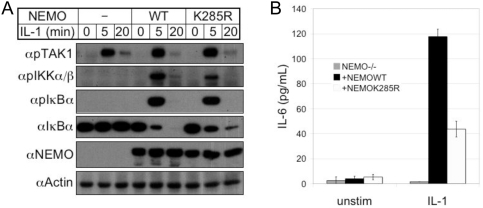
Ubiquitination of NEMO by TRAF6 is partially required for IL-1-mediated activation of NFκB. (A) NEMO-deficient fibroblasts retrovirally-rescued with wild-type or K285R NEMO were treated as indicated with IL-1β, then lysed and subjected to immunoblotting against the activated phosphorylated forms of TAK1, IKKα/β, and IκBα. (B) NEMO-deficient fibroblasts retrovirally-rescued with WT or K285R NEMO were left untreated or treated for 12 hours with IL-1β. Supernatants were collected and assayed by ELISA for IL-6 production. Values are normalized to crystal violet assays of the cell culture plates.

Lack of a requirement for TRAF6 autoubiquitination in TRAF6 RING finger-dependent signaling suggests that alternative physiologic targets of TRAF6 E3 ligase activity must exist. To confirm the relevance of TRAF6-mediated NEMO ubiquitination to physiologic NFκB activation, we retrovirally rescued NEMO-deficient fibroblasts with wild-type or K285R NEMO and treated with IL-1. We found that despite normal TAK1 activation, and nearly normal phosphorylation of IκBα, activation of IKKα/β was considerably diminished, and degradation of IκBα was slightly reduced (Fig. 6A). To determine whether these signaling defects affected NFκB-dependent cytokine production, we assayed supernatant from the IL-1-treated fibroblasts for IL-6 production, and found a greater than 50% reduction in fibroblasts rescued with K285R as compared to wild-type NEMO. These observations suggest not only that TRAF6-mediated ubiquitination of NEMO is physiologically relevant, but also that TRAF6-dependent signaling likely involves targets of TRAF6 E3 ligase activity beyond TRAF6 itself.

## Discussion

The non-conventional RING finger E3 ligase activity of TRAF6 is critical for activating downstream signaling in the key inflammatory and developmental pathways IL-1R and RANK, respectively. TRAF6 autoubiquitination is commonly used as an activation marker for RING finger activity, and it is currently held that K63-linked ubiquitin chains on TRAF6 serve as a docking platform for downsteam mediators like TAK1 [1]. In this context, the requirement for TRAF6 autoubiquitination per se has not been formally demonstrated. We attempted to do so by divorcing the RING finger function of TRAF6 from its ubiquitin substrate function. Surprisingly, we found that in the absence of TRAF6 autoubiquitination, IL-1- and RANKL-dependent signaling remained intact in the presence of a functional RING finger domain. Retrovirally-rescued TRAF6-deficient fibroblasts elaborated IL-6 in response to IL-1, and retrovirally-rescued TRAF6-deficient BMM developed into osteoclasts in response to RANKL, both independently of TRAF6 autoubiquitination. TRAF6 autoubiquitination was also found to be dispensable for TRAF6 RING finger-dependent ubiqutination of NEMO. Further, we found that neither TRAF6 autoubiquitination, nor the RING finger domain were required for TRAF6 interaction with the TAK1 complex.

Our initial efforts to identify specific ubiquitin acceptor lysines on TRAF6 revealed that while we could detect reduced TRAF6 autoubiquitination in some cases, these reductions were not accompanied by reduced TRAF6 function. This was true even for TRAF6 K124, the recently identified acceptor site reported to be essential simultaneously for TRAF6 autoubiquitination and signaling [22], (Fig. S3). By taking the dramatic step of mutating all lysines on TRAF6 to arginine in order to ablate all potential acceptor sites, we were forced to acknowledge certain caveats. First, some residues may be important for protein stability. While lysine ablation did not affect expression of N-terminal TRAF6ΔK(1-358), full-length ΔK protein expression was dramatically reduced. Regardless, any loss in protein stability was not sufficient to affect the signaling functions examined. Second, we may have inadvertently abrogated the capacity of TRAF6 to undergo other lysine-dependent modifications, such as acetylation, methylation, sumoylation, or K48-linked degradative ubiquitination. Published data indicates a negative regulatory mechanism for TRAF6 via IFNγ-induced K48-linked ubiquitination and proteasomal degradation [30]. Our observation that lysine ablation in the N-terminus of TRAF6 (Fig. 2) leads to increased NFκB and AP-1 activity may indicate interference with a similar type of negative regulation. Additional efforts are required to fully characterize the effects of this mutational pattern on TRAF6 protein stability and turnover.

**Figure 7:**
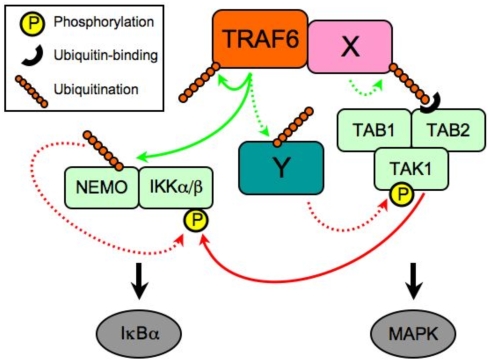
A revised model for TRAF6-dependent activation of the MAPK and IKK signaling pathways in the absence of TRAF6 autoubiquitination. Upon receptor engagement, K63-linked ubiquitin chains are affixed to TRAF6, at least in part through the E3 ubiquitin ligase activity of the TRAF6 RING finger domain. Both TRAF6 autoubiquitination and the TRAF6 RING finger domain appear dispensable for recruitment of the TAB1-TAB2-TAK1 complex. Previous reports indicate this interaction to be dependent on the ability of the TAB2 ubiquitin-binding domain to attach to K63-linked ubiquitin chains [17], suggesting an unidentified factor “X”, which interacts with TRAF6 independently of autoubiquitination, may serve as the binding partner for TAB2. At the same time, recruitment of TAK1 to TRAF6 is insufficient for TAK1 activation, which specifically requires TRAF6 RING finger activity. An additional unknown factor “Y” may be indicated, in this case to serve as a substrate(s) for TRAF6-mediated K63-linked ubiquitination. Upon being ubiquitinated, factor “Y” would promote TAK1 phosphorylation, leading to MAPK and IKKα/β activation. TRAF6-mediated activation of the IKKα/β complex is only partially dependent on TAK1 [31], but exhibits an absolute requirement for the regulatory component IKKγ (NEMO) [32], [33]. NEMO ubiquitination on K285 by TRAF6 is required for optimal activation of IKKα/β. RING finger-dependent activation of TAK1 and IKKα/β by TRAF6 might involve K63-linked ubiquitination of numerous targets, which together form a signaling complex sufficient to meet activation thresholds. TRAF6 autoubiquitination may, in fact, be required in the absence of alternative ubiquitin substrates, or in as yet untested signaling systems lacking robust redundancy. This schematic underscores the point that in contrast to the absolute requirement for TRAF6 RING finger E3 ligase activity in mediating downstream signaling, K63-linked ubiquitination of TRAF6 should be viewed as a marker of activation.

Cumulatively, our findings regarding the genetic dispensability of TRAF6 autoubiquitination lead us to a revised model for TRAF6-mediated activation of the NFκB and MAPK pathways (Fig. 7). Upon receptor engagement, K63-linked ubiquitin chains are affixed to TRAF6, at least in part through the E3 ubiquitin ligase activity of the TRAF6 RING finger domain. Both TRAF6 autoubiquitination and the TRAF6 RING finger domain appear dispensable for recruitment of the TAB1-TAB2-TAK1 complex. Previous reports indicate this interaction to be dependent on the ability of the TAB2 ubiquitin-binding domain to attach to K63-linked ubiquitin chains [17], suggesting an unidentified factor “X”, which interacts with TRAF6 independently of autoubiquitination, may serve as the binding partner for TAB2. At the same time, recruitment of TAK1 to TRAF6 is insufficient for TAK1 activation, which specifically requires TRAF6 RING finger activity. An additional unknown factor “Y” may be indicated, in this case to serve as a substrate(s) for TRAF6-mediated K63-linked ubiquitination. Upon being ubiquitinated, factor “Y” would promote TAK1 phosphorylation, leading to MAPK and IKKα/β activation. TRAF6-mediated activation of the IKKα/β complex is only partially dependent on TAK1 [31], but exhibits an absolute requirement for the regulatory component IKKγ (NEMO) [32], [33]. NEMO ubiquitination on K285 by TRAF6 is required for optimal activation of IKKα/β. RING finger-dependent activation of TAK1 and IKKα/β by TRAF6 might involve K63-linked ubiquitination of numerous targets, which would together form a signaling complex sufficient to meet activation thresholds.

Recent efforts by various groups have identified additional targets of TRAF6 E3 ubiquitin ligase activity, including IRF-7 downstream of TLRs 7–9 and LMP1 [34], [35], IRF-5 downstream of TLRs 7 and 9 [36], NRIF, TrkA and TrkB, associated with NGFR [16], [37], [38], and RIP2 and NEMO downstream of NOD [7], [39]. Intriguingly, in the IL-1R pathway, it has recently been demonstrated that IRAK-1, which interacts with TRAF6 through a TRAF6 interaction motif, is modified by K63-linked ubiquitination in a TRAF6 RING finger-dependent manner [40], [41]. Further, it has been shown that NEMO contains a K63-specific ubiquitin-binding domain, required for IL-1-mediated IKK activation, which mediates interaction with ubiquitinated IRAK-1, but not ubiquitinated TRAF6 [40], [41]. Examining our data in this context suggests that IRAK-1 and TRAF6 ubiquitination may be redundant, or that recruitment of TAK1 via TAB2/3 ubiquitin-binding may be mediated through ubiquitination of IRAK-1 (and/or one of the alternative TRAF6 targets listed above) rather than TRAF6. TRAF6 autoubiquitination may, in fact, be required in the absence of alternative ubiquitin substrates, or in other signaling systems lacking robust redundancy. Crosstalk between multiple TRAF6-associated scaffolding systems, specifically TRAF6-IRAK-NEMO in the IL-1R/TLR pathway and TRAF6-p62 in the atypical PKC pathway [42], may also create redundancies which obviate TRAF6 autoubiquitination. Further, while we have focused here on TRAF6-mediated activation of the TAK1 pathway, it should be noted that the kinase MEKK3 governs an alternate TRAF6-dependent signaling pathway, for which the role of TRAF6 autoubiquitination remains unclear [25].

In this study we have undertaken a limited survey of the ever-expanding array of signaling pathways serviced by TRAF6. Assessment of the physiologic role of TRAF6 autoubiquitination may require cell-type specific examination of signals emanating from TLRs, NLRs, additional TNFRSF members, TGFβR, IL-17R and IL-25R, and others. However, our data demonstrate that TRAF6 autoubiquitination is clearly dispensable for TRAF6 function in two key signaling pathways, and suggest that TRAF6-mediated K63-linked ubiquitination instead targets multiple relevant protein substrates during activation. Hopefully these findings will contribute specifically to defining the mechanism of TRAF6 function, and generally to elucidating the role of non-conventional ubiquitination in signal transduction.

## Materials and Methods

### Cell Lines, Reagents, and Antibodies

HEK293 cells were purchased from the American Type Culture Collection (ATCC). The Plat-E retroviral packaging cell line, NEMO-deficient fibroblasts, and TAK1-deficient fibroblasts were kindly provided by T. Kitamura (University of Tokyo), Michael Karin (UCSD, San Diego), and Sankar Ghosh (Yale University), respectively. TRAF6-deficient fibroblasts were derived from lung or peritoneal tissue of E14.5 fetuses. IL-1α and IL-1β were purchased from R&D Systems (Minneapolis, MN). Soluble RANKL was purified from insect cells and M-CSF was kindly provided by David Fremont (Washington University, St. Louis, MO). TRAP solution substrate and Coumermycin A were purchased from Sigma (St. Louis, MO). Antibodies specific for FLAG (M2 and M5) and Myc (9E12) were from Sigma (St. Louis, MO); for phospho-TAK1 (T187), phospho-p38, phospho-IκBα, phospho-IKKα/β, IκBα, p38, JNK, TAK1, IKK, and NEMO were from Cell Signaling (Danvers, MA); for TRAF6 was from MBL (Tokyo, Japan); for phospho-JNK was from BD Biosciences (Franklin Lakes, NJ); and for Ubiquitin was from Millipore (Billerica, MA).

### Plasmids

Murine TRAF2, TRAF5, and TRAF6 were cloned into the NotI and BamHI sites of CMV2-pFLAG (Sigma; St. Louis, MO). Gyrase B-pEF was kindly provided by Jun-ichiro Inoue (University of Tokyo). TRAF6 N-terminus-Gyrase B fusion constructs were made by cloning Gyrase B into an EcoRI site downstream of and in frame with TRAF6(1-358) and the BamHI site. Various TRAF6 and NEMO point mutations were generated by PCR using the Stratagene (La Jolla, CA) Quick Change site-directed mutagenesis kit. Murine splenic cDNA was used to clone TAK1, TAB1, TAB2, and MEKK3 by PCR into pcr2.1 using Zero Blunt Topo, and subcloned into pcDNA3.1-MycHis (Invitrogen; Carlsbad, CA). FLAG-NEMO and FLAG-IKKβEE were kindly provided by Michael May (University of Pennsylvania). FLAG-ASK1 was kindly provided by Eui-Ju Choi (Korea University).

### Cell Culture, Transfections and Retroviral Transduction

5×10^6^ 293T and plat-E cells were cultured in DMEM supplemented with 10% FBS, penicillin/streptomycin (all purchased from Invitrogen; Carlsbad, CA) in 10 cm tissue culture-treated dishes. Cells were transfected with 15 µL Superfect (Qiagen; Venlo, Netherlands) plus DNA cocktails for 2 hours, then washed with fresh medium, and cultured for an additional 36 hours before harvesting cells or supernatants. Retroviral supernatants were harvested after 36 hours transfection, mixed 1∶1 with fresh medium, and added to cells to be transduced with 5 µg/mL polybrene (Sigma; St. Louis, MO) for 6 hours. 24 hours post-transduction, infected cells were replated in fresh medium containing 2 µg/mL puromycin for 2 days selection.

### Immunoprecipitation and Western Blotting

Cells were harvested with ice-cold PBS and scraping, pelleted, and lysed with 1 mL lysis buffer (20 mM HEPES buffer pH 7.5, 150 mM NaCl, 10% (w/v) glycerol, protease inhibitor cocktail (Roche; Basel, Switzerland), 2 mM sodium ortho-vanadate, 2 mM NaF, 100 ng/mL Calyculin A (Cell Signaling; Danvers, MA), 1.25 mg/mL N-Ethyl Malemide (Sigma; St. Louis, MO)) with 1.0% (w/v) Triton-×100 or 0.5% NP-40 (for immunoprecipitation). Lysates were incubated on ice for 20 minutes, and clarified by centrifugation at 20,000×g for 10 minutes. Whole cell extracts were aliquotted, added to 6× SDS loading buffer and boiled. Extracts for *in vivo* ubiquitination analysis were first boiled with 10% SDS for 10 minutes, then diluted 10-fold with lysis buffer. Otherwise, extracts for IP were transferred to 50 µL anti-FLAG or anti-Myc agarose (Sigma, St. Louis, MO), and incubated while rocking at 4°C for 3 hours. IP agarose beads were washed 3× with lysis buffer, resuspended in SDS loading buffer and boiled. Samples were run on 10% SDS-PAGE and transferred to PVDF membrane (Millipore; Billerica, MA). Blots were probed with primary antibodies in 5% milk dissolved in PBS with 0.1% Tween-20, followed by secondary anti-rabbit-HRP or anti-mouse-HRP (Promega; Madison, WI). Western blots were incubated with ECL substrate (Pierce; Rockford, IL) and exposed to film.

### *In Vitro* Ubiquitination

Assays were performed in 30 µL reactions with buffer containing 300 mM HEPES at pH 7.2, 50 mM MgCl_2_ and 2 mM dithiothreitol. Additional components included 0.15 µg recombinant rabbit E1 activating enzyme (Boston Biochem) and 0.6 µg recombinant mouse Ubc13/Uev1a conjugating complex (Boston Biochem). 2.5 µg recombinant ubiquitin (Boston Biochem) containing all lysines, K48 only, or K63 only was added as indicated. 20 mM ATP was added as indicated. Ubiquitin E3 ligase/substrate was prepared as a 50 µL IP of cell lysates containing the indicated version of FLAG-tagged TRAF6, from which 15 µL was added to a 2× reaction cocktail. Reactions were incubated for 1 hour at 30°C with 450 rpm shaking, and then washed 3× with buffer alone to separate unincorporated components from IP beads containing the TRAF6 substrate.

### Osteoclast Cultures

Bone marrow stem cells were flushed from femurs and tibias of chimeric mice reconstituted with liver cells harvested from wild-type or TRAF6-deficient E14.5 fetuses, and cultured with M-CSF (30 ng/ml) for 5 days in α-minimal essential medium (α-MEM) containing 10% fetal bovine serum. Attached cells were transduced with retrovirus on day 2 and replated on day 3 for 48 hours selection with puromycin (2 µg/mL). Selected bone marrow macrophages (BMM) were replated on day 5 in 96-well plates (2×10^4^/well) with fresh medium supplemented with M-CSF (30 ng/ml) and RANKL (100 ng/ml) for 4 days (medium and cytokines refreshed after 2 days) to induce osteoclastogenesis. For TRAP solution assays, cells were fixed with 10% formalin, permeabilized with methanol/acetone (1∶1), dried, and then incubated with TRAP substrate solution for 30 minutes at room temperature. Substrate solution was mixed 2∶1 with 1N NaOH, and quantified by 405 nm absorbance. Osteoclast cell counts were determined by enumerating TRAP+ cells larger than 100 µm in diameter with more than three nuclei in each well.

### Reporter Assays

HEK293 cells were transfected in triplicate with test plasmids plus 50 ng luciferase reporter plasmid and 10 ng β-Galactosidase control plasmid in 6-well plates. Cells were harvested and lysed with Tropix Lysis Buffer (Applied Biosystems; Foster City, CA). Lysates were freeze-thawed 3× before clarification by centrifugation. 15 µL of lysate was mixed with Tropix substrate for 30 minutes, added to 100 µL Tropix accelerator and β-Galactosidase assayed colorimetrically. 15 µL of lysate was mixed with 100 mL luciferase substrate (Promega; Madison, WI), and assayed colorimetrically for reporter activity. Reporter activity was normalized to β-Galactosidase levels.

### Mass Spectrometry

Proteins from gel bands were digested with modified trypsin, and peptides analyzed by microflow reverse phase-HPLC/MS/MS with a ThermoFinnigan LCQ Deca XP LTQ-OrbitrapXL Mass Spectrometer with Nano-capillary HPLC operated in positive-ion mode at a flow-rate of 250 nl/min and a 75 µm (ID)×15 µm×5 cm C18 column. SEQUEST (ThermoFinnigan, San Jose, CA) software was used to identify all MS/MS data for NEMO modified lysine residues corresponding to an ubiquitin modification (GG, 114.1 Da).

## Supporting Information

Figure S1 In vitro autoubiquitination of TRAF6 is RING finger-dependent . 293T cells were transfected with FLAG-TRAF6 or FLAG-TRAF6 C85A/H87A, immunoprecipitated with FLAG, and lysates subjected to in vitro ubiquitination in the presence or absence of ATP using biotinylated recombinant ubiquitin. Unmodified TRAF6 was detected by immunoblotting with anti-TRAF6 and polyubiquitination was detected with streptavidin-HRP (SA-HRP).
(0.63 MB TIF)

Figure S2 TAK1 T187 is required for optimal IL-1-dependent NFκB and MAPK signaling. TAK1-deficient fibroblasts were retrovirally-rescued with empty vector, wild-type TAK1, or TAK1 T187A were treated as indicated with IL-1β, then lysed and subjected to immunoblotting against the activated phosphorylated forms of IκBα and JNK.
(1.05 MB TIF)

Figure S3 TRAF6 K124 is not required for IL-1-dependent NFκB and MAPK signaling. TRAF6-deficient fibroblasts were retrovirally-rescued with the indicated full-length versions of TRAF6, including one containing a single lysine to arginine mutation at K124, and treated as indicated with IL-1β, then lysed and subjected to immunoblotting against the activated phosphorylated forms of IκBα or JNK.
(1.13 MB TIF)

## References

[1] Chen ZJ (2005). Ubiquitin signalling in the NF-kappaB pathway.. Nat Cell Biol.

[2] Lowe EL, Doherty TM, Karahashi H, Arditi M (2006). Ubiquitination and de-ubiquitination: role in regulation of signaling by Toll-like receptors.. J Endotoxin Res.

[3] Laine A, Ronai Z (2005). Ubiquitin chains in the ladder of MAPK signaling.. Sci STKE.

[4] Deng L, Wang C, Spencer E, Yang L, Braun A (2000). Activation of the IkappaB kinase complex by TRAF6 requires a dimeric ubiquitin-conjugating enzyme complex and a unique polyubiquitin chain.. Cell.

[5] Wang C, Deng L, Hong M, Akkaraju GR, Inoue J (2001). TAK1 is a ubiquitin-dependent kinase of MKK and IKK.. Nature.

[6] Ye H, Arron JR, Lamothe B, Cirilli M, Kobayashi T (2002). Distinct molecular mechanism for initiating TRAF6 signalling.. Nature.

[7] Abbott DW, Yang Y, Hutti JE, Madhavarapu S, Kelliher MA (2007). Coordinated regulation of Toll-like receptor and NOD2 signaling by K63-linked polyubiquitin chains.. Mol Cell Biol.

[8] Lee MS, Kim YJ (2007). Signaling pathways downstream of pattern-recognition receptors and their cross talk.. Annu Rev Biochem.

[9] Maezawa Y, Nakajima H, Suzuki K, Tamachi T, Ikeda K (2006). Involvement of TNF receptor-associated factor 6 in IL-25 receptor signaling.. J Immunol.

[10] Schwandner R, Yamaguchi K, Cao Z (2000). Requirement of tumor necrosis factor receptor-associated factor (TRAF)6 in interleukin 17 signal transduction.. J Exp Med.

[11] Sorrentino A, Thakur N, Grimsby S, Marcusson A, von Bulow V (2008). The type I TGF-beta receptor engages TRAF6 to activate TAK1 in a receptor kinase-independent manner.. Nat Cell Biol.

[12] Wu H, Arron JR (2003). TRAF6, a molecular bridge spanning adaptive immunity, innate immunity and osteoimmunology.. Bioessays.

[13] Yamashita M, Fatyol K, Jin C, Wang X, Liu Z (2008). TRAF6 mediates Smad-independent activation of JNK and p38 by TGF-beta.. Mol Cell.

[14] Takaoka A, Yanai H, Kondo S, Duncan G, Negishi H (2005). Integral role of IRF-5 in the gene induction programme activated by Toll-like receptors.. Nature.

[15] Takayanagi H (2005). Mechanistic insight into osteoclast differentiation in osteoimmunology.. J Mol Med.

[16] Geetha T, Jiang J, Wooten MW (2005). Lysine 63 polyubiquitination of the nerve growth factor receptor TrkA directs internalization and signaling.. Mol Cell.

[17] Kanayama A, Seth RB, Sun L, Ea CK, Hong M (2004). TAB2 and TAB3 activate the NF-kappaB pathway through binding to polyubiquitin chains.. Mol Cell.

[18] Singhirunnusorn P, Suzuki S, Kawasaki N, Saiki I, Sakurai H (2005). Critical roles of threonine 187 phosphorylation in cellular stress-induced rapid and transient activation of transforming growth factor-beta-activated kinase 1 (TAK1) in a signaling complex containing TAK1-binding protein TAB1 and TAB2.. J Biol Chem.

[19] Haglund K, Dikic I (2005). Ubiquitylation and cell signaling.. Embo J.

[20] VanDemark AP, Hill CP (2002). Structural basis of ubiquitylation.. Curr Opin Struct Biol.

[21] Baud V, Liu ZG, Bennett B, Suzuki N, Xia Y (1999). Signaling by proinflammatory cytokines: oligomerization of TRAF2 and TRAF6 is sufficient for JNK and IKK activation and target gene induction via an amino-terminal effector domain.. Genes Dev.

[22] Lamothe B, Besse A, Campos AD, Webster WK, Wu H (2007). Site-specific Lys-63-linked tumor necrosis factor receptor-associated factor 6 auto-ubiquitination is a critical determinant of I kappa B kinase activation.. J Biol Chem.

[23] Walsh MC, Kim N, Kadono Y, Rho J, Lee SY (2006). Osteoimmunology: interplay between the immune system and bone metabolism.. Annu Rev Immunol.

[24] Chung JY, Park YC, Ye H, Wu H (2002). All TRAFs are not created equal: common and distinct molecular mechanisms of TRAF-mediated signal transduction.. J Cell Sci.

[25] Huang Q, Yang J, Lin Y, Walker C, Cheng J (2004). Differential regulation of interleukin 1 receptor and Toll-like receptor signaling by MEKK3.. Nat Immunol.

[26] Mercurio F, Zhu H, Murray BW, Shevchenko A, Bennett BL (1997). IKK-1 and IKK-2: cytokine-activated IkappaB kinases essential for NF-kappaB activation.. Science.

[27] Mochida Y, Takeda K, Saitoh M, Nishitoh H, Amagasa T (2000). ASK1 inhibits interleukin-1-induced NF-kappa B activity through disruption of TRAF6-TAK1 interaction.. J Biol Chem.

[28] Sakurai H, Nishi A, Sato N, Mizukami J, Miyoshi H (2002). TAK1-TAB1 fusion protein: a novel constitutively active mitogen-activated protein kinase kinase kinase that stimulates AP-1 and NF-kappaB signaling pathways.. Biochem Biophys Res Commun.

[29] Perkins ND (2006). Post-translational modifications regulating the activity and function of the nuclear factor kappa B pathway.. Oncogene.

[30] Takayanagi H, Ogasawara K, Hida S, Chiba T, Murata S (2000). T-cell-mediated regulation of osteoclastogenesis by signalling cross-talk between RANKL and IFN-gamma.. Nature.

[31] Shim JH, Xiao C, Paschal AE, Bailey ST, Rao P (2005). TAK1, but not TAB1 or TAB2, plays an essential role in multiple signaling pathways in vivo.. Genes Dev.

[32] Rudolph D, Yeh WC, Wakeham A, Rudolph B, Nallainathan D (2000). Severe liver degeneration and lack of NF-kappaB activation in NEMO/IKKgamma-deficient mice.. Genes Dev.

[33] Yamaoka S, Courtois G, Bessia C, Whiteside ST, Weil R (1998). Complementation cloning of NEMO, a component of the IkappaB kinase complex essential for NF-kappaB activation.. Cell.

[34] Kawai T, Sato S, Ishii KJ, Coban C, Hemmi H (2004). Interferon-alpha induction through Toll-like receptors involves a direct interaction of IRF7 with MyD88 and TRAF6.. Nat Immunol.

[35] Ning S, Campos AD, Darnay BG, Bentz GL, Pagano JS (2008). TRAF6 and the three C-terminal lysine sites on IRF7 are required for its ubiquitination-mediated activation by the tumor necrosis factor receptor family member latent membrane protein 1.. Mol Cell Biol.

[36] Balkhi MY, Fitzgerald KA, Pitha PM (2008). Functional regulation of MyD88 activated IRF-5 by K63-linked polyubiquitination.. Mol Cell Biol.

[37] Geetha T, Kenchappa RS, Wooten MW, Carter BD (2005). TRAF6-mediated ubiquitination regulates nuclear translocation of NRIF, the p75 receptor interactor.. Embo J.

[38] Jadhav T, Geetha T, Jiang J, Wooten MW (2008). Identification of a consensus site for TRAF6/p62 polyubiquitination.. Biochem Biophys Res Commun.

[39] Yang Y, Yin C, Pandey A, Abbott D, Sassetti C (2007). NOD2 pathway activation by MDP or Mycobacterium tuberculosis infection involves the stable polyubiquitination of Rip2.. J Biol Chem.

[40] Conze DB, Wu CJ, Thomas JA, Landstrom A, Ashwell JD (2008). Lys63-linked polyubiquitination of IRAK-1 is required for interleukin-1 receptor- and toll-like receptor-mediated NF-kappaB activation.. Mol Cell Biol.

[41] Windheim M, Stafford M, Peggie M, Cohen P (2008). Interleukin-1 (IL-1) induces the Lys63-linked polyubiquitination of IL-1 receptor-associated kinase 1 to facilitate NEMO binding and the activation of IkappaBalpha kinase.. Mol Cell Biol.

[42] Moscat J, Diaz-Meco MT, Wooten MW (2007). Signal integration and diversification through the p62 scaffold protein.. Trends Biochem Sci.

