# Supplementary figures and images for: TRAF6 Autoubiquitination-Independent Activation of the NFκB and MAPK Pathways in Response to IL-1 and RANKL
Source: PLoS One. 2008 Dec 29;3(12):e4064. doi: 10.1371/journal.pone.0004064 (PMC2603309; doi:10.1371/journal.pone.0004064)

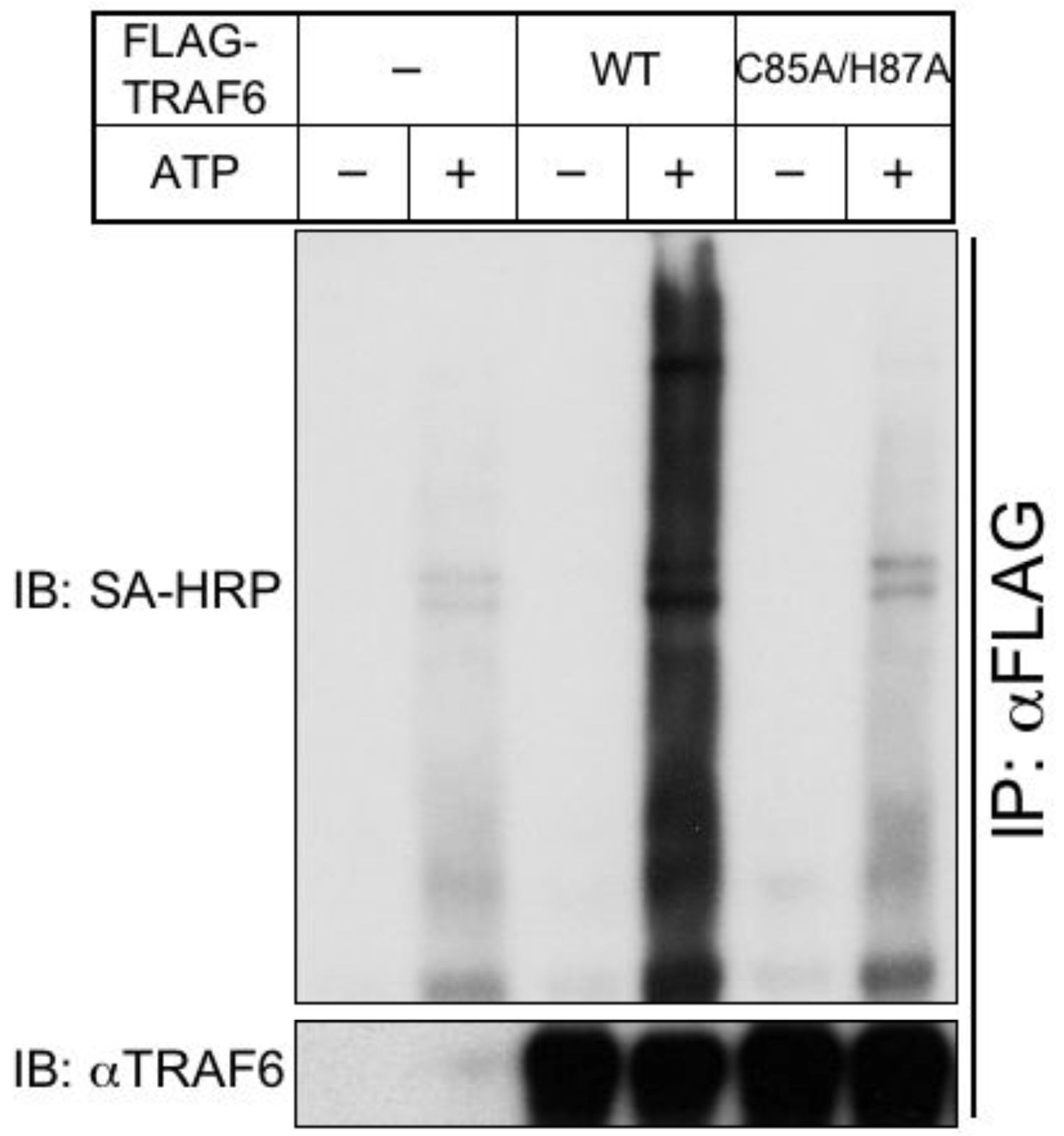

Supplement: Figure S1 — In vitro autoubiquitination of TRAF6 is RING finger-dependent . 293T cells were transfected with FLAG-TRAF6 or FLAG-TRAF6 C85A/H87A, immunoprecipitated with FLAG, and lysates subjected to in vitro ubiquitination in the presence or absence of ATP using biotinylated recombinant ubiquitin. Unmodified TRAF6 was detected by immunoblotting with anti-TRAF6 and polyubiquitination was detected with streptavidin-HRP (SA-HRP). (0.63 MB TIF) [file pone.0004064.s001.tif]

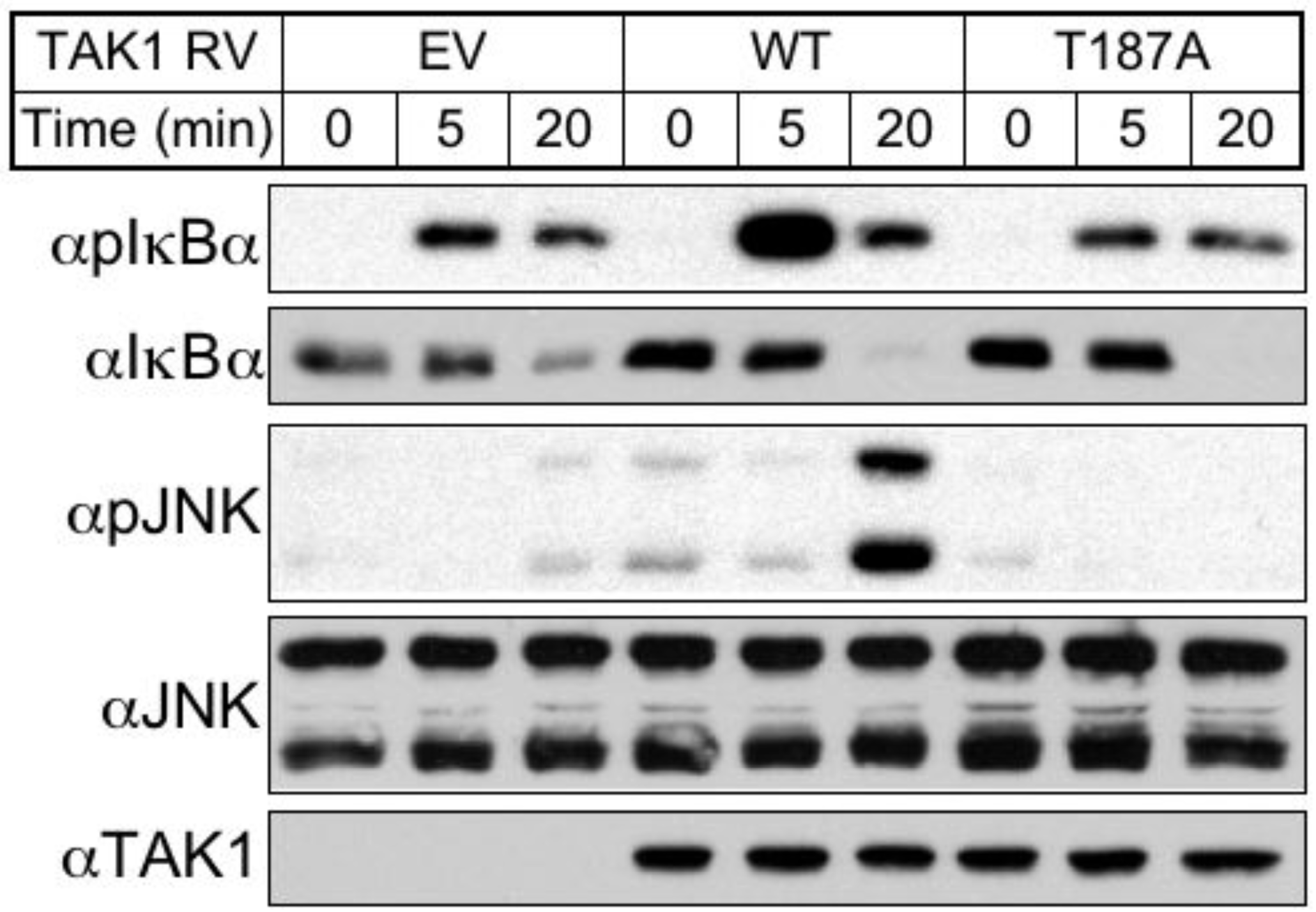

Supplement: Figure S2 — TAK1 T187 is required for optimal IL-1-dependent NFκB and MAPK signaling. TAK1-deficient fibroblasts were retrovirally-rescued with empty vector, wild-type TAK1, or TAK1 T187A were treated as indicated with IL-1β, then lysed and subjected to immunoblotting against the activated phosphorylated forms of IκBα and JNK. (1.05 MB TIF) [file pone.0004064.s002.tif]

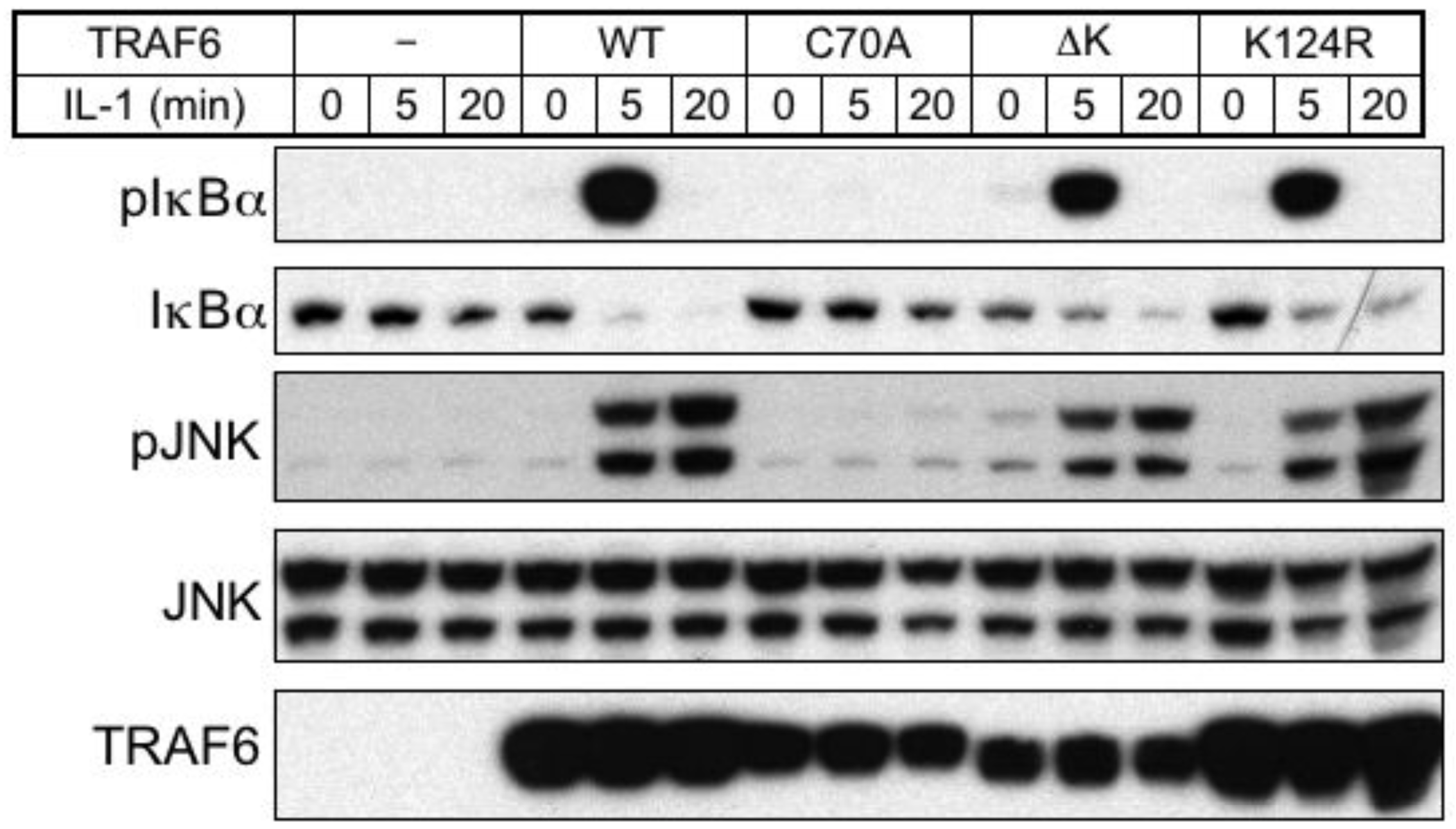

Supplement: Figure S3 — TRAF6 K124 is not required for IL-1-dependent NFκB and MAPK signaling. TRAF6-deficient fibroblasts were retrovirally-rescued with the indicated full-length versions of TRAF6, including one containing a single lysine to arginine mutation at K124, and treated as indicated with IL-1β, then lysed and subjected to immunoblotting against the activated phosphorylated forms of IκBα or JNK. (1.13 MB TIF) [file pone.0004064.s003.tif]
